# Novel Microwave-Assisted Synthesis of Poly(D,L-lactide): The Influence of Monomer/Initiator Molar Ratio on the Product Properties

**DOI:** 10.3390/s100505063

**Published:** 2010-05-20

**Authors:** Ljubisa Nikolic, Ivan Ristic, Borivoj Adnadjevic, Vesna Nikolic, Jelena Jovanovic, Mihajlo Stankovic

**Affiliations:** 1 Faculty of Technology, University of Nis, Bulevar oslobodjenja 124, 16000 Leskovac, Serbia; E-Mails: nvesna@yahoo.com (V.N.); mstankovic_99@yahoo.com (M.S.); 2 Faculty of Technology, University of Novi Sad, Cara Lazara 1, 21000 Novi Sad, Serbia; E-Mail: ivancekaris@yahoo.com; 3 Faculty for Physical Chemistry, University of Belgrade, Studentski trg 12-16, 11000 Belgrade, Serbia; E-Mails: adnadjevic@nadlanu.com (B.A.); jelenaj@ffh.bg.ac.yu (J.J.)

**Keywords:** poly(D,L-lactide), novel microwave synthesis, ring-opening polymerization (ROP)

## Abstract

Poly(D,L-lactide) synthesis using tin(II) 2-ethylhexanoate initiated ring-opening polymerization (ROP) takes over 30 hours in bulk at 120 °C. The use of microwave makes the same bulk polymerization process with the same initiator much faster and energy saving, with a reaction time of about 30 minutes at 100 °C. Here, the poly(lactide) synthesis was done in a microwave reactor, using frequency of 2.45 GHz and maximal power of 150 W. The reaction temperature was controlled via infra-red system for in-bulk-measuring, and was maintained at 100 °C. Different molar ratios of monomer and initiator, [M]/[I], of 1,000, 5,000 and 10,000 were used. The achieved average molar masses for the obtained polymers (determined by gel permeation chromatography) were in the interval from 26,700 to 112,500 g/mol. The polydispersion index was from 2.436 to 3.425. For applicative purposes, the obtained material was purified during the procedure of microsphere preparation. Microspheres were obtained by spraying a fine fog of polymer (D,L-lactide) solution in tetrahydrofuran into the water solution of poly(vinyl alcohol) with intensive stirring.

## 1. Introduction

Polymers based on lactic acid deserve great attention because they decompose by hydrolysis in the human body into nontoxic metabolites. Among the many applications found for these polymers in medicine, it is worth mentioning: a fracture fixer [1,2], surgical cord for the inner lesions suture [2–6], various implants [7,8] and material for target therapy or controlled release of medications [9–20]. The traditional method of poly(lactide) (PLA) synthesis required rigorous conditions: a high vacuum, long polymerization times and the consumption of great quantities of energy, using metal or metal oxide as a catalyst to speed up the reaction and minimize the pyrolysis by reducing the temperature [21–29].

Polymerization of lactide has the same basic approach:

– Re-crystallization of lactide monomer in order to eliminate possible impurities;
– Drying of the monomer and ampoules for polymerization, because lactide hydrolyzes in the presence of even traces of moisture;
– Filling of ampoules with the monomer mixture and the initiator, and sealing under extreme vacuum;
– Polymerization process at high temperature, usually from 100 to 130 °C, sometimes even up to 280 °C, for a duration of 20 to 30 hours, sometimes over 50 hours;
– Precipitation of the obtained polymer from the solution by means of a non-solvent to eliminate the residual monomer and initiator;
– Drying under vacuum.

**Figure 1.**
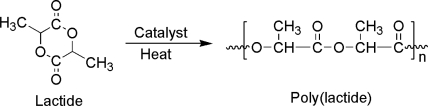
Scheme of poly(lactide) synthesis.

The poly(lactide) synthesis is carried out by ring-opening polymerization according to the scheme given in the Figure 1.

The difficulties of poly(lactide) synthesis can be successfully overcome by microwave heating. Microwave radiation has numerous advantages compared to conventional heating: homogeneous heating of the whole volume of the reaction mixture, high transfer energy per unit of time, improved yield, the possibility of the process acceleration and synthesis without using great quantity of the solvent. Polymerization assisted by microwave provides a new approach for enhancing polymer properties as well as economic advantages through energy saving and accelerated product development. Animated by numerous successes in the organic synthesis field, the use of microwaves enables a drastic reduction of polymerization time, to only 5–30 minutes, while obtaining polymers of high molar mass at the same time. For example, super absorbing resin was synthesized by using microwaves in only six minutes [30].

The power of microwaves of 150 W has been used in the synthesis of poly(D,L-lactide), which is several times lower than the power used in other polymer synthesis processes, using up to 800 W [31].

The homogeneous nature of microwave heating eliminates local overheating at the reaction walls, which can lead to side products. Therefore, microwave-irradiated reactions are not only faster, but proceed with higher purity and, consequently, higher yields. In an industry where time is money, the dramatic rate acceleration and increased purity and yields of microwave assisted reactions make them attractive for high-produced polymers.

To develop a technique of microwave-assisted polymerization of D,L-lactide, to efficiently and easily prepare poly(D,L-lactide) with high molecular weight, the ring opening polymerization of D,L-lactide by microwave irradiation under atmosphere was investigated. Both lactic acid and its oligomer are polar molecules, so they can absorb microwave energy to increase the temperature.

## 2. Experimental Section

### 2.1. Materials

D,L-Lactide (3,6-dimethyl-1,4-dioxane-2,5-dione), (98% purity) was from Sigma-Aldrich Wisconsin. Tin(II) 2-ethylhexanoate (Stannous octoate), (95% purity), density 1,251 g/mL at 25 °C was from Sigma-Aldrich Wisconsin. Chloroform and methanol were high-performance liquid chromatography grade. Other solvents, toluene, tetrahydrophurane (THF), and water were of reagent grade. All solvents were purchased from Merck Chemical Co. Poly(vinyl alcohol) (PVA, 88 mol% hydrolyzed, M_w_ 25,000) was purchased from Polysciences, Inc. (Warrington, PA, USA).

Three mol ratios of monomer and initiator were used for the synthesis: [M]/[I] = 1,000, 5,000 and 10,000. Composition of reaction mixtures for the synthesis in microwave reactor are shown in Table 1.

### 2.2. Microwave-Assisted Synthesis of PLLA

Dry D,L-lactide (5 g, 34.7 mmol), pre-crystallized from methanol, was placed in evaporating bowls, 14.05, 2.81 or 1.41 mg tin(II) 2-ethylhexanoate (34.7, 6.9 and 3.47 mol) was added with 1 cm^3^ dry, fresh distilled toluene. The mixture was homogenized, and then toluene was evaporated at 60 °C in vacuum for 12 h. The reaction mixture was then removed into glass ampoule and closed under reduced pressure. Polymerization was performed in a “Discover” focus microwave reactor, CEM Corporation, Matthews, NC, USA. The frequency and the power applied were 2.45 GHz and 150 W, respectively. The temperature regulation was carried out by infrared mass measuring system and maintained at 100 °C.

### 2.3. Poly(D,L-lactide) Microsphere Preparation

After polymerization, the polymer was precipitated by methanol from the chloroform solution to purify it from residual monomer and initiator. Poly(D,L-lactide) were dissolved in 10 mL tetrahydrofuran to provide concentration of 2 to 4% wt/vol. The solution was then sprinkled into a 200 mL aqueous solution containing 0.5% wt/vol poly(vinyl alcohol) (PVA). The mixture was stirred on a hot plate magnetic stirrer to form a stable emulsion system at room temperature (25 ± 2 °C). Stirring was continued for 3 hour at 65 °C to allow the evaporation of tetrahydrofuran and the formation of solid micro-spheres. Microspheres were filtered, washed with distilled water, and dried until no weight loss was observed.

### 2.4. Characterization of Obtained Polymers and Microspheres

Fourier transform infrared spectrum, FTIR, was recorded by Bomem Hartmann & Braun MB-series. Samples were milled with KBr (0.5 mg of the sample with 150 mg of KBr) and formed tablets under vacuum press. Recording was performed in the wave band range from 400 to 4,000 cm^−1^.

The molecular weight of obtained polymers was determined by gel permeation chromatography, GPC, using Agilent 1100 Series system with refractive index, RID 1200, and diode array, DAD, 1200 (recording at 212 nm) detectors. Used column ZORBAX PSM 300, 250 × 6.2 mm, 5 μm, covered molecular mass range 3 × 10^3^–3 × 10^5^ g/mol and operated at temperature 25 °C. Tetrahydrofuran used as eluent (flow 1 cm^3^/min). Sample injection volume was 10 μl. The average molar masses, *M_n_*, *M_w_* and poly(D,L-lactide) polydispersivity index Q were determined by software Agilent ChemStation for LC and GPC. Poly(styrene) standards were used to make calibration curve: 10.000 g/mol (Mw = 10.640, Mn = 9.940, Mp = 10.860, Q = 1.07, FLUKA), 100.000 g/mol (Mw = 94.900, Mn = 89.300, Mp = 89.400, Q = 1.06, FLUKA), 300.000 g/mol (Mw = 319.000, Mn = 305.000, Mp = 321.000, Q = 1.05, FLUKA).

The morphologies of the microspheres were observed using a scanning electron microscope (SEM, JEOL JSM–5300, Japan). The microspheres were vacuum dried at room temperature, mounted onto brass stubs and sputter-coated with gold in an argon atmosphere using JEOL JFC–1100 ion sputter.

## 3. Results and Discussion

**Figure 2.**
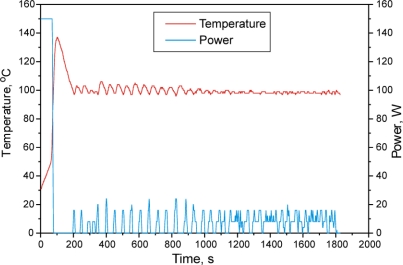
Temperature and microwave radiation power dependence on reaction time (sample MWS-6) for (poly(D,L-lactide) synthesis in bulk.

This work concerned the tin(II) 2-ethylhexanoate initiated synthesis of poly(D,L-lactide). Figure 2 shows the temperature and the applied power of the reaction mixture as dependent on the reaction time. D,L-Lactide readily absorbs the microwaves, having as a result a fast temperature increase in the first 80 seconds. After the start of the reaction, heat is released due to the exothermic effect of the polymerization reaction (since almost all of the initiator is included in the reaction). The temperature (140 °C) rises above the appointed value (100 °C) although the microwave radiation is automatically switched off, as the program of the microwave reactor is set at maintaining the temperature at 100 °C. The applied power of 150 W at the beginning of the reaction becomes zero after reaching the appointed temperature (in about 1.5 minutes). In the sequel of the polymerization reaction, power of only about 20 W is applied to maintain the temperature, in pulses of several tens of seconds with intermittent pauses of similar duration. The graph shows that the beginning of the polymerization occurs at 70 °C, that the absorption of microwaves decreases with the increase of the polymer content in the reaction mixture and that the highest absorption is that of pure monomer.

The change of reaction mixture temperature from the period of 3–20 minutes shows that the microwave absorption is still present and that there is still some monomer in the reaction mixture to be polymerized, hence the temperature leaps from the polymerization reaction exothermicity. Namely, every intermittent temperature leap follows the automatic turning off of microwave radiation. After 20 minutes the temperature change becomes insignificant and it is maintained constant only through the microwaves absorption, but there are no more temperature leaps or exothermal processes in the reaction mixture, indicating that the monomer conversion into polymer is complete.

**Figure 3.**
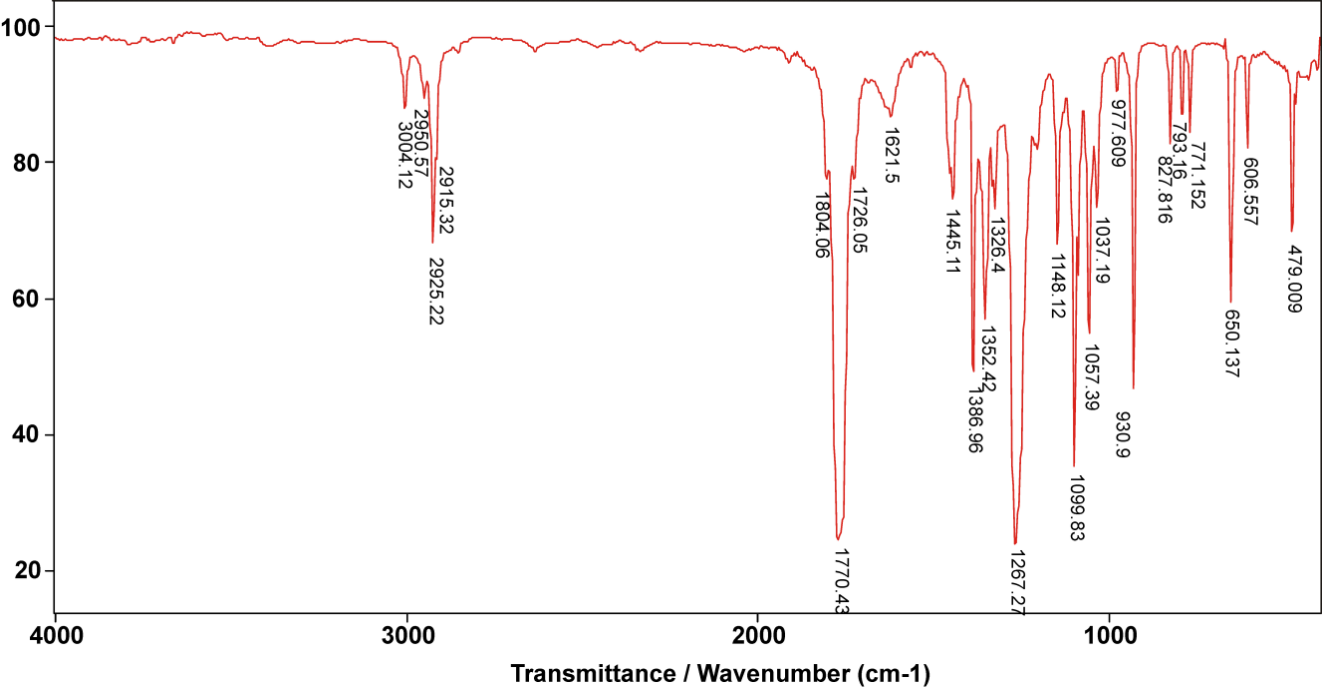
FTIR spectrum of monomer D,L-lactide.

The molecular structures of synthesized polymers were confirmed by FTIR methods. FTIR spectrum of the monomer D,L-lactide (Figure 3) shows bands at 2,915.32 and 2,950.57 cm^−1^ from symmetric and asymmetric valence vibrations of C-H, respectively. Bands at 2,925.22 and 3,004.12 cm^−1^ originate from symmetric and asymmetric valence vibrations of C-H from CH_3_, respectively. In the FTIR spectrum of the monomer D,L-lactide, bands also appear at 1,267.27 cm^−1^ (asymmetric valence vibrations of C-O-C in the lactonic ring), 1,099.83 cm^−1^ (symmetric valence vibrations of C-O-C in the lactonic ring), 1,770.43 cm^−1^ (cyclic dilactone C = O valence vibration), 1,445.11 and 1,386.96 cm^−1^ (asymmetric and symmetric bending vibration of C-H from CH_3_, respectively) and 930.9 cm^−1^ (COO ring breathing mode).

**Figure 4.**
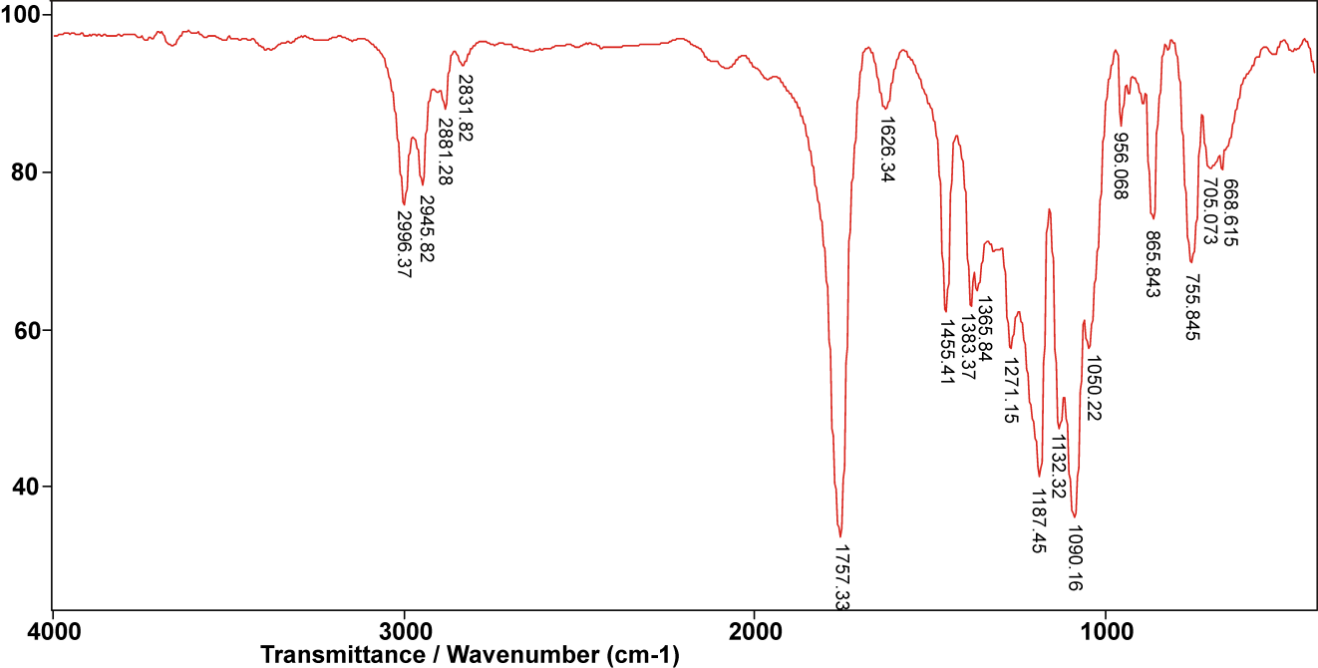
FTIR spectrum of poly(D,L-lactide).

**Figure 5.**
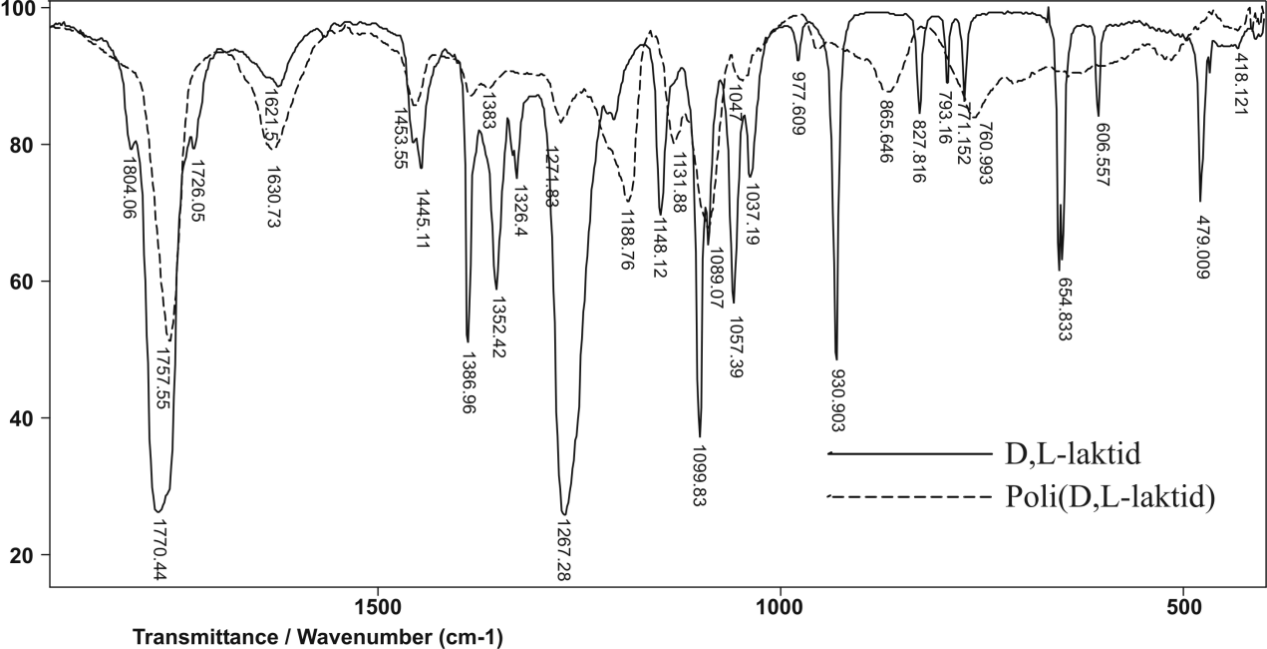
FTIR spectrum of monomer D,L-lactide and obtained poly(D,L-lactide) in the wave band range from 400 to 2,000 cm^−1^.

In the FTIR spectrum of poly(D,L-lactide), with the monomer/initiator ratio of 1/5,000, obtained for 15 minutes, bands were present at 2,831.82 and 2,945.82 cm^−1^ from symmetric and asymmetric valence vibrations of C-H, respectively (Figure 4). At 2,881.28 and 2,996.37 cm^−1^, bands were from symmetric and asymmetric valence vibrations of C-H from CH_3_. Asymmetrical valence vibrations of C-O-C of the aliphatic chain were shifted at 1,187.45 cm^−1^, and symmetrical valence vibrations of C-O-C of the aliphatic chain 1,090.16 cm^−1^, compared with bands at 1,276 and 1,099 cm^−1^, which appeared in monomer D,L-lactide, Figure 5. Accompanying bands at 1,757.33 cm^−1^ (valence vibration of C═O of aliphatic ester), 1,455.41 and 1,383.37 cm^−1^ (asymmetric and symmetric bending vibration of C-H from CH_3_, respectively), 1,271.15 cm^−1^ (the overlap C-H bending vibration and C-O-C stretching vibration) were also detected.

**Figure 6.**
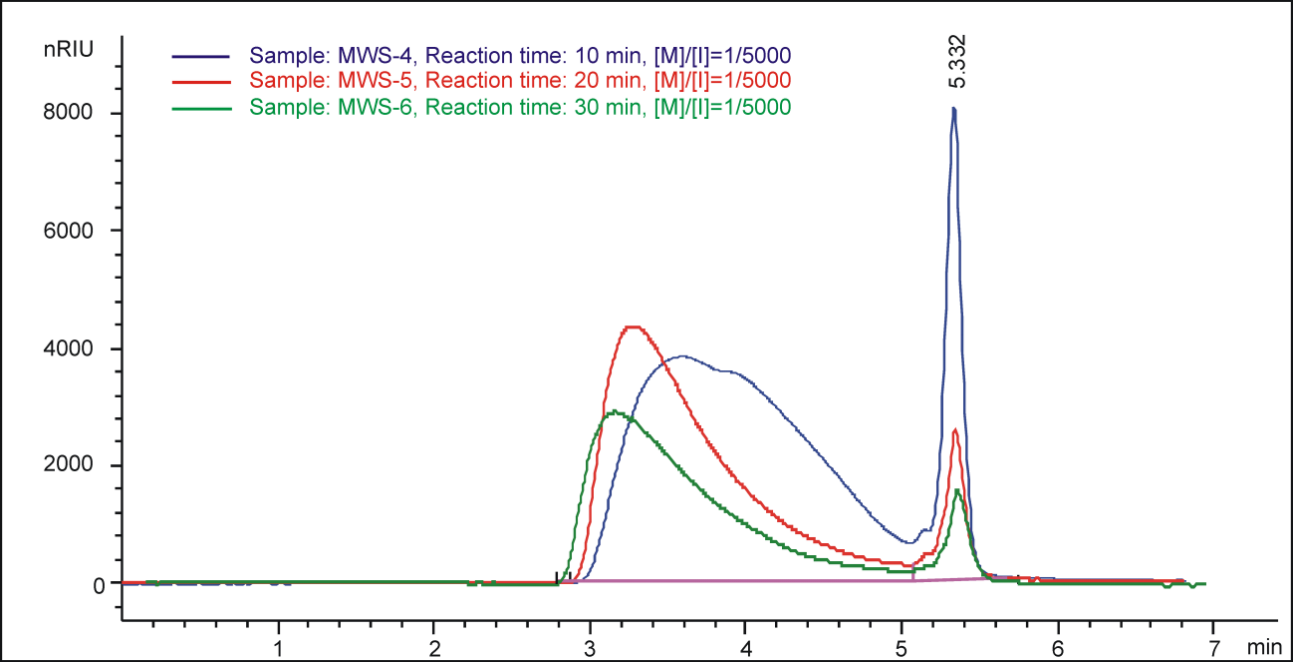
The signal at RID detector in function of eluation volume for samples with the same monomer/initiator ratio, 1/5,000, obtained for different reaction times.

**Figure 7.**
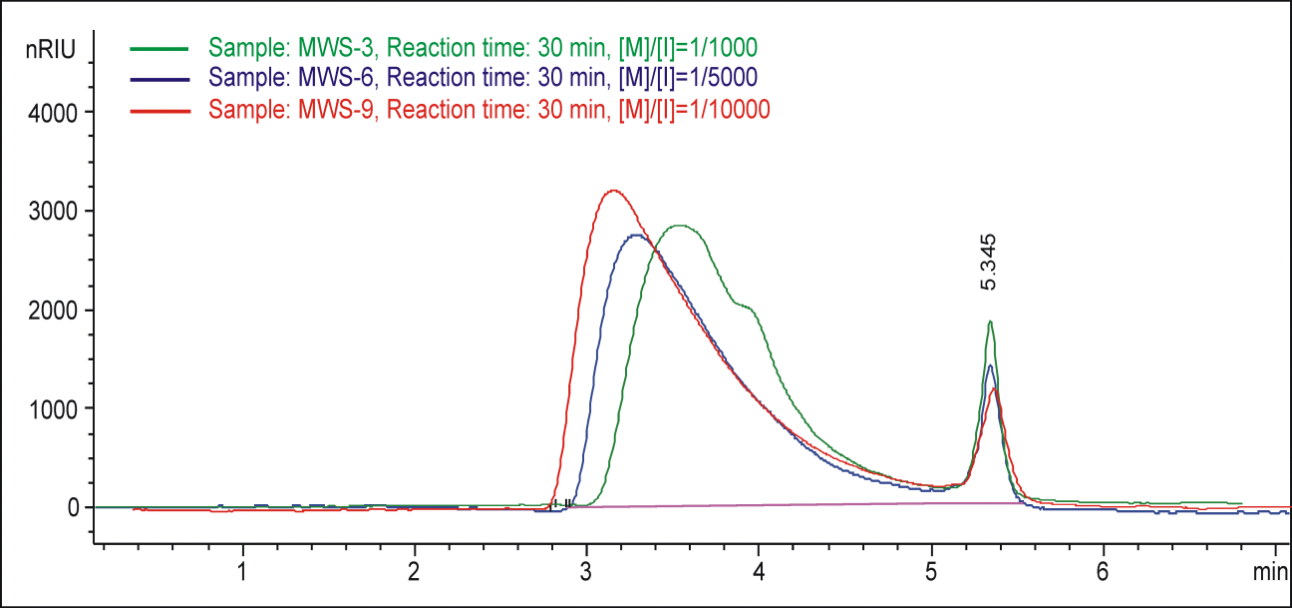
The signal at RID detector in function of eluation volume for samples with different monomer to initiator, [M]/[I], ratio.

The GPC curves of synthesized polymer are shown in Figures 6 and 7. Unreacted monomer was found in the profile for poly(D,L-lactide) synthesized by microwaves, but with increasing reaction time a decreased quantity of unreacted monomers was observed (retention time 5.33 minutes), Figure 6. Figure 7 shows GPC curves for poly(D,L-lactide)s polymerized at the same time with different monomer/initiator ratios. As expected, it can be concluded that with decreasing initiator content, the molecular mass of obtained polymers increases. Peaks from unreacted monomers decreased with decreasing initiator content.

Table 2 shows the values of mean molar masses *M_n_*, *M_w_* and polydispersivity index Q, for poly(D,L-lactide) synthesized using microwaves as the function of polymerization time and monomer/initiator mol ratio [M]/[I]. The values of mean molar mass and the polydispersivity index were observed to increase with the increase of reaction duration. These values were also increased with the increase of monomer/initiator ratio.

**Figure 8.**
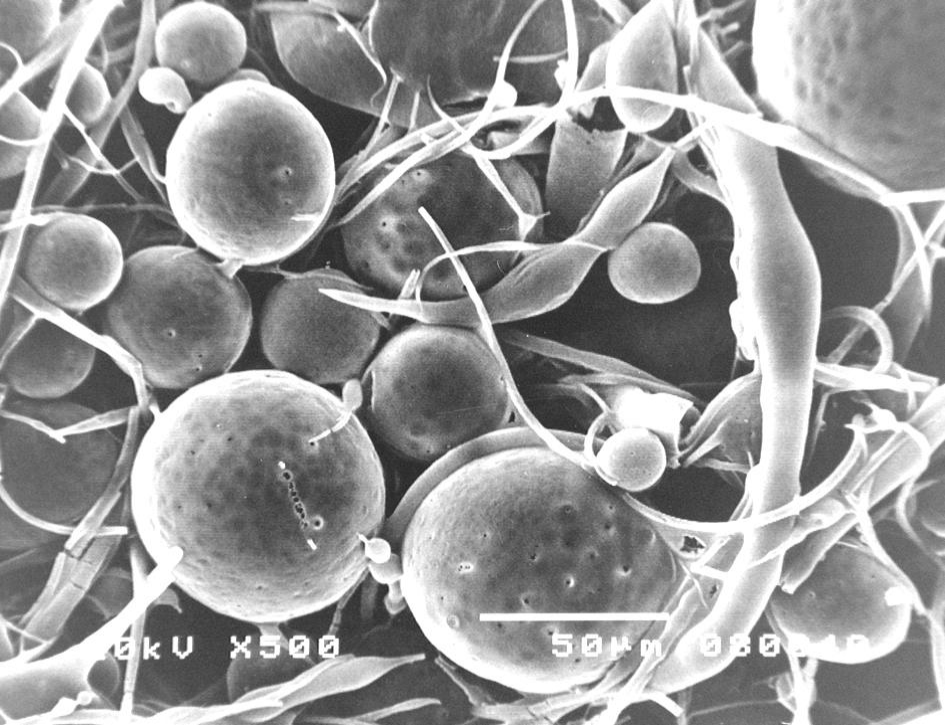
SEM micrograph of poly(D,L-lactide) microspheres; magnified 500 X, bar = 50 μm.

Figure 8 shows the SEM image of poly(D,L-lactide) spheres obtained by spraying a fine fog of poly(D,L-lactide) solution in tetrahydrofuran into the water solution of poly(vinyl alcohol) with intensive stirring.

Such microspheres can be used as polymer matrices for the production of devices for controlled release of medicinal substances, since the diameter of the microspheres is appropriate for phagocytosis by macrophages.

## 4. Conclusions

The reduction of poly(D,L-lactide) synthesis duration and energy consumption by using microwaves enables a more economical production. The microwave synthesis requires more energy during the first few seconds only to obtain uniform and intensive initiation. The polymerization reaction course is readily supervised by monitoring the temperature of the reaction mixture by infra-red sensors and the applied power of microwave radiation. poly(D,L-lactide) could be synthesized effectively by microwave-assisted ring opening polymerization using tin(II)2-ethylhexanoate as a initiator. This microwave assisted polymerization was much faster than the literature data for polymerization heated by a conventional oil bath under similar reaction conditions. The monomer/initiator ratios had a strong influence on the molecular masses and polydispersity of obtained polymers. A higher monomer/initiator ratio resulted in polymers with higher molar masses and lower polydispersity, Q. At a reaction temperature of 100 °C, the prolonged microwave irradiation time showed a significant effect on the increasing polymer molar mass.

From SEM imagine of microspheres it was concluded that the technique provides uniform sized spheres. The size of the obtained microspheres was about 50 μm.

## Figures and Tables

**Table 1.** Composition of reaction mixtures for the synthesis in microwave reactor (molar concentration of monomer [M] and [I] initiator).

Sample	[M]/[I]	m_M_, g	m_C_, mg

MWS-1 MWS-2 MWS-3	1/1,000	5	14.05
MWS-4 MWS-5 MWS-6	1/5,000	5	2.81
MWS-7 MWS-8 MWS-9	1/10,000	5	1.41

m_M_ - the mass of the monomer

m_C_ - the mass of the initiator

**Table 2.** Mean molar masses *M_n_*, *M_w_* and polydispersivity index Q for poly(D,L-lactide) synthesized using microwaves, as the function of the polymerization time and monomer/initiator [M]/[I] mol ratio.

Sample	Reaction time, min	[M]/[I]	*M_n_*, g/mol	*M_w_*, g/mol	Q	Yield (%)

MWS-1	10	1/1,000	35,820	108,033	3.016	68
MWS-2	20	1/1,000	40,982	127,126	3.102	81
MWS-3	30	1/1,000	59,483	203,729	3.425	89
MWS-4	10	1/5,000	26,724	78,461	2.936	81
MWS-5	20	1/5,000	42,470	131,359	3.093	83
MWS-6	30	1/5,000	102,321	287,111	2.806	83
MWS-7	10	1/10,000	32,627	79,479	2.436	87
MWS-8	20	1/10,000	62,075	164,498	2.650	89
MWS-9	30	1/10,000	112,542	309,940	2.754	95
